# Evaluating a specialist primary care service for patients experiencing homelessness: a qualitative study

**DOI:** 10.3399/bjgpopen20X101049

**Published:** 2020-07-08

**Authors:** Emily Clark, Emily Player, Tara Gillam, Sarah Hanson, Nicholas Steel

**Affiliations:** 1 Norwich Medical School, University of East Anglia, Norwich, UK; 2 City Reach Health Services, Norfolk Community Health and Care NHS Trust, Norwich, UK; 3 Faculty of Medicine and Health Sciences, University of East Anglia, Norwich, UK

**Keywords:** Inequalities, Patient perspectives, Mental health, Homeless persons, People experiencing homelessness, Primary health care, General practice

## Abstract

**Background:**

People experiencing homelessness (PEH) often experience poor health, multimorbidity, and early mortality and experience barriers to accessing high quality health care. Little is known about how best to provide specialist primary care for these patients.

**Aim:**

To evaluate the health care provided to patients experiencing homelessness who were seen in a specialist primary care service.

**Design & setting:**

A qualitative evaluation of a city centre primary healthcare service for excluded and vulnerable people, such as rough sleepers, who find it difficult to visit mainstream GP services.

**Method:**

Data on patient characteristics and service use were extracted from primary care records using electronic and free-text searches to provide context to the evaluation. Semi-structured interviews with 11 patients and four staff were used to explore attitudes and experiences.

**Results:**

Patients had high needs compared with the general population. Patients valued continuity of care, ease of access, multidisciplinary care, and person-centred care. Staff were concerned that they lacked opportunities for reflection and learning, and that low clinical capacity affected service safety and quality. Staff also wanted more patient involvement in service planning.

**Conclusion:**

PEH’s complex health and social problems benefited from a specialist primary care service, which is thought to reduce barriers to access, treat potentially challenging patients in a non-judgmental way, and provide personal continuity of care in order to develop trust.

## How this fits in

Previous studies recommended evaluation of specialist models of primary care to understand how best to provide services for excluded groups, such as patients experiencing homelessness. This study supports the role of specialist service as adding unique qualities to primary care. The Faculty for Homeless and Inclusion Health (FIH) framework of values can guide service providers, commissioners, and staff in provision of services, service evaluation, and quality improvement. The learning from this study can help clinicians to understand how to deliver primary care to patients experiencing homelessness by, for example, reducing barriers to access, treating patients in a non-judgmental way, and placing importance on continuity of care.

## 



## Introduction

People experiencing homelessness (PEH) often experience poor health, multimorbidity, and early mortality,^1^ and rough sleepers die 30 years earlier than the general population on average.^2^ PEH may experience barriers to accessing high quality care, including inflexible services and appointment systems and negative staff attitudes, leading to exclusion from mainstream services.^3^ In addition to poorer health outcomes, their care directly translates into an increased burden for the health and social care sector; PEH in England are admitted to secondary care 3.2 times as often as the general population.^4^

Inclusion health is a service, research, and policy agenda that aims to prevent and redress health and social inequities among the most vulnerable and excluded populations.^5^ A synthesis of 77 systematic reviews of health and social interventions for inclusion health in 2018^5^ detailed evidence for psychosocial interventions, case management, disease prevention, and schemes for housing and social determinants of health. It recommended research on specialist and mainstream models of primary care to understand how best to provide services for excluded groups, and that future studies should evaluate both patient and provider experiences of primary care provision for patients experiencing homelessness.^5^

This study aimed to evaluate the care provided to patients experiencing homelessness who were seen in a specialist primary care service, with reference to national standards for inclusion health.

### Study setting

The setting was a bespoke, city centre primary healthcare service providing healthcare services for people who find it difficult to visit mainstream GP services, such as rough sleepers, sex workers, and vulnerable migrants. Staff included GPs, nurses, administrative workers, and support workers. In total, 609 patients were registered at the clinic (as of September 2018), of which 470 were classed as ‘experiencing homelessness’; these patients were the focus of this study. Those classed as ‘experiencing homelessness’ includes patients living in temporary accommodation and hostels as well as rough sleepers and ‘sofa-surfers’. The clinic offers additional support such as visiting in-reach drug and alcohol support workers, Hepatitis C clinic, needle exchange, shower, and some basic necessities such as donated clothes.

## Method

Routinely collected service use, demographic, and Read code data on patient and practice characteristics were extracted from the practice SystmOne electronic records. Patient characteristics were also manually collected from 207 patient medical records, including free-text and Read code data. The 207 records were a randomly selected subsection, generated by Microsoft Excel (2016), from the whole caseload. These data provided background context to the patient characteristics.^6^

### Design and data

Care given at the service met the Medical Research Council’s definition of ‘highly complex’ in that: it had several interacting components; success was affected by the behaviour of those delivering or receiving the intervention; and great variability existed in the individuals served and outcomes expected.^7^

Administrative data and primary care outcomes can miss the full impact of complex interventions.^8^ Therefore face-to-face, semi-structured interviews^9^ were used to explore patients’ experiences of the care provided and the perspectives of staff. Interviews were used to allow rapport to be established and to encourage honest dialogue.^10^

**Figure 1.**
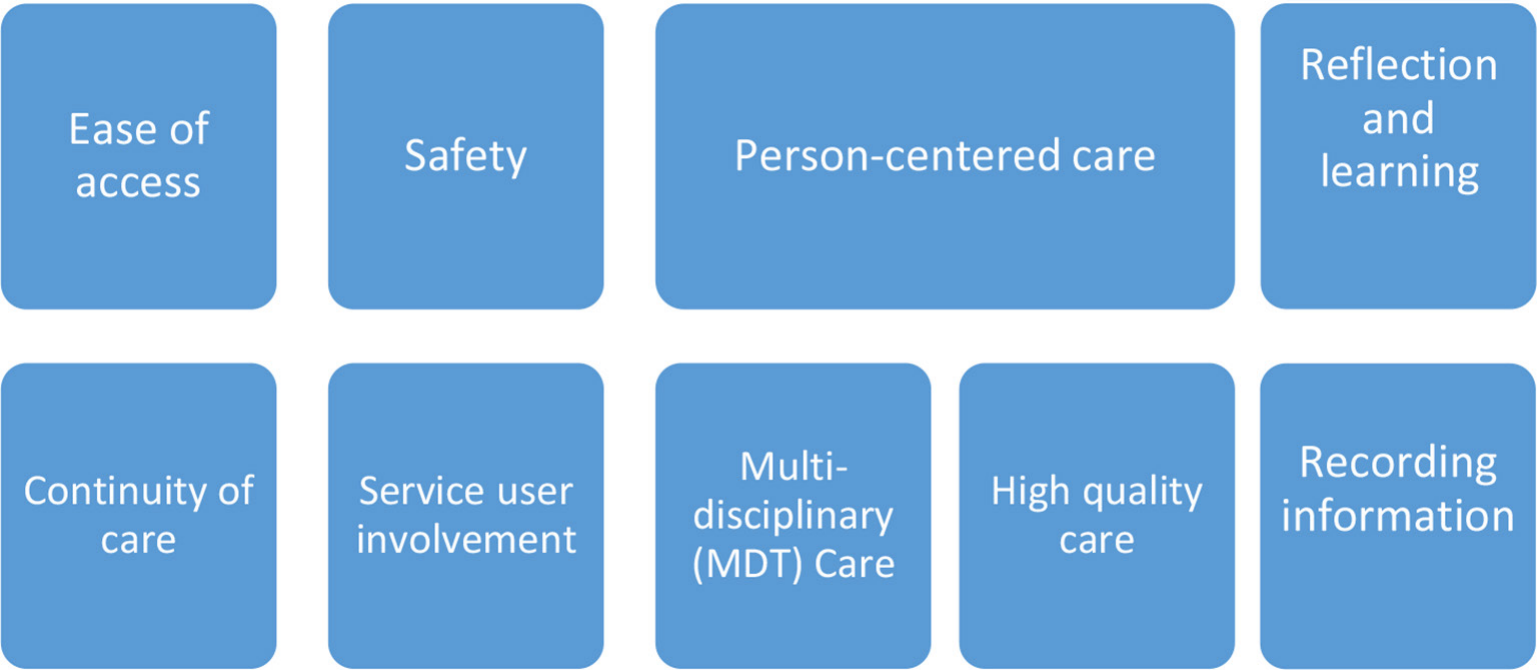
Faculty for Homeless and Inclusion Health key values for providing care

Separate topic guides were used for staff and patient interviews. Topics were agreed by the research team (TG, EP, and EC) to elucidate the participants' views on how the service fulfilled the standards of the FIH (Figure 1); patients were asked direct questions on how easy they found it to get an appointment (value of ease of access), whether they felt safe attending the service (value of safety), and whether they felt that they could give feedback to the service to influence patient experiences (service user involvement).

### Recruitment

Clinical staff suggested patients to be interviewed so as to represent the range of patients who use the services, including rough sleepers and patients living in hostels. Patients who had been registered at the practice for at least 6 months were selected. Due to the often unpredictable engagement patterns of patients accessing the service, a pragmatic approach to interviewing was taken and interviewers selected those they were able to get into contact with (by phone, for those who had phones), or those who were available after their clinical appointment (for those who did not have phones).^11^ The lead researcher did not interview her own patients.^12^ Staff were included if they had worked for the service for ≥1 year and were selected using purposive sampling to include administration, nursing, and medical staff.

Interviews were carried out by the lead researcher and another female GP with experience of working with patients experiencing homelessness. All interviews were completed at the surgery and took 20–60 minutes. They were audio-recorded and transcribed by the main author.

### Ethical considerations

Patients and staff were informed that taking part in the research was entirely optional and that declining would not affect their care or employment. Informed written consent was obtained from patients and staff by signing a statement of understanding. No personally identifiable information was included in the transcripts or made available outside of the study team. Shop vouchers worth £10 each were given to patients taking part in the study, but to avoid coercion this was not discussed until after patients had consented and interviews had taken place. Standard clinical governance feedback routes within the provider organisation were in place in the case of issues arising regarding patient or staff safety. Given the vulnerable nature of this cohort of patients, if traumatic narratives were revealed this was dealt with sensitively and appropriate follow-up with their chosen GP was facilitated.

### Analysis

The lead researcher transcribed patient and staff interviews and used NVIVO software (vresion 12) for data organisation. These were fully anonymised and then analysed by the lead investigator (EC) using the principles of framework analysis,^13^ which is a suitable method for the management and analysis of qualitative data in applied health research with mixed data from different participants and vignettes. Framework analysis was designed to facilitate evaluation of health and social care interventions and allows comparison across, between, and within cases. It enabled the analysis to focus on the core issues explored in the interviews, while offering the flexibility to explore new themes. The FIH is a multidisciplinary body which has produced standards for commissioners and service providers to follow when planning, commissioning, and providing health care for PEH and other excluded groups.^14^ Data were mapped onto the FIH values for providing care (Figure 1); these are continuity of care, ease of access, multidisciplinary care, person-centred care, high quality care, safety of service, service user involvement, and reflection and learning.

## Results

The mean age of patients was 36 years. This is important because in the UK, the average age at death is 44 years for men experiencing homelessness and 42 years for women experiencing homelessness.^15^ Of these patients, 74% were male and 26% were female; 52% had a criminal record; and 44% had experienced severe trauma (which could include domestic violence and/or childhood abuse).

Patients experiencing homelessness had high needs compared with the general population. The Read code dataset in this evaluation showed that 8% of patients had a long-term physical health problem, whereas bespoke data collection of 207 patient records showed that 30% did. This discrepancy is due to inadequate Read coding; for example, some patients were prescribed regular inhalers without a Read code diagnosis of chronic obstructive pulmonary disease (COPD) or asthma. Thirteen per cent of patients at the clinic were known to have Hepatitis B or C infection, compared with 0.5% of the general population.^16^ 87% of patients in this evaluation had a mental health condition compared with 17% of the general population.^17^ All mental health diagnoses (psychotic disorders, depression, and anxiety) were significantly higher in this practice population than in the general population. 68% of this practice population were on prescribed medication compared with 48% of the general population.^18^

Patients also had higher rates of addictions. 70% of this practice population had used an illicit drug compared with 8.4% of the general adult population in England and Wales.^18^ 77% of this practice population smoke, compared with 14.9% of the adult population in England.^18^ This may be an underestimate of rates of smoking in this practice population, as smoking is only screened for at the initial health check, which is not always completed. 52% of this practice population misuse alcohol compared with 1.35% of the general population.^19^

### Patient perspectives

Interviews were completed with 11 patients. Patients were predominantly male, and had a wide range of clinical and social problems (Table 1).

**Table 1.** Participant characteristics for 11 patients interviewed

Demographics	Frequency, *n*
Male	9
Female	2
Age range, years	21—62
**Health information**	
Mental health problem	11
Long-term physical health problem	8
Trimorbidity (mental health/addiction/physical health)	8
Alcohol dependency	4
Substance misuse	8
Opiate substitution therapy	7
>4 Long-term medications	7
>1 'High risk' medications	7
Infectious disease	5
Malnutrition (treated)	2
**Circumstances**	
Sex worker	2
Offending history	9
Poor education/literacy	5
Unemployed	10
Own flat	3
Street homelessness	2
Hostel	2
Living in van	1
B&B	1
Sofa surfing	2
Supported by third sector organisations	3
Claiming benefit	11
**Personal history**	
In care as a child	3
Childhood abuse	3
Breakdown of personal relationship	8
Traumatic event	11

B&B = Bed and breakfast.

#### Ease of access

The interviews also gave insight into the reasons why individuals struggled to engage with mainstream primary care services. For example, many had previous negative experiences with services, which further inhibited trust of professionals:

*‘* *The mental health team cut me off because I didn’t have a home* *.* *'* (Participant [P]11)

The practice improved access and attendance for patients by a proactive approach to missed appointments:

‘*I come here every week. If I miss my appointment I get a bloody phone call* *!* *’* (P4)

Tolerance of behaviour in the waiting room was also felt to improve access for patients compared with mainstream primary care:

‘*A* *couple of times, you have had people that are cantankerous, that are difficult, that raise their voices* … *But the guys here seem to know exactly what to do and seem to be on top of that.*’ (P9)

The only disadvantage of a specialist service was that patients on a journey of recovery found having contact with old acquaintances in the waiting room difficult:

‘*It’s a bit of a bummer bumping into the odd person. And they are still using* [drugs] *or whatever and it’s like* ‘‘*get away from me* *''* *. But that’s the only difficulty.*’ (P3)

#### Continuity of care

Continuity of care was important for patients, especially in terms of developing an honest and open relationship with their GP, which improved compliance with treatment and engagement:

‘I have always said, I prefer sticking with Dr ****. If I had any choice, I would stick with her. It’s probably the longest relationship I have had in my life.’ (P8)

This continuity of care had benefits for the patient and doctor, and facilitated patients' engagement when relapsing, perhaps in terms of addiction:

*‘I have been coming here that long, my doctor gets me, knows where I am at, when I am going off at the deep end and when I am doing well. So she has insight into that. And that’s built up over time.*’ (P7)

There was also a sense that if this continuity of care was interrupted, it could lead to disengagement:

‘*I am comfortable here, I know everyone. I hate change. When change happens, the way I deal with is to walk away from it. Which isn’t always good I know.*’ (P6)

#### Multidisciplinary team (MDT) care

The practice’s multidisciplinary approach to providing care — including outreach services led by support workers and nurses, integrated drug and alcohol service, and mental health and Hepatitis C clinics⁠ — was recognised:

‘*This is the best place in Norwich to come to, if you are using* [drugs] *or wanting to get off or whatever you need. They are a lot more useful than other doctors.*’ (P10)

#### Person-centred care

This study also showed the challenge of the complex interplay of emotions;

*‘No other surgery would take me, I was abusive, loud and didn’t give a toss.’* (P4)

behaviour;

‘*I was losing my marbles again, and was doing heroin again and started climbing the walls, and this is when I started cutting myself again. I was getting all erratic, I was breaking down and going in and out of A&E* [Accident & Emergency].’ (P4)

and history of trauma;

*‘I was sexually abused by my father so I was taken off my parents when I was 5. Then I was in care for 11 years and sexually abused there in a private care home.’* (P8)

A person-centred approach, incorporating staff listening, showing humanity, and being ‘non-judgmental’, was valued by many patients:

‘*Here* [they] *are always supportive, they always listen to the person. I have been registered to another surgery. They weren’t really listening to me, they were just writing stuff on a computer, they just put me on a load of medication, and it made me more suicidal.*’ (P8)

A further aspect to person-centred care was addressing patients' basic needs. This appears to be a key mechanism to enable patients to engage and would suggest that only once the basic physiological needs for shelter, nutrition, and warmth are addressed can deeper issues such as addiction or mental health problems be looked at:

*‘* *I have a shower here, they give me a clean set of clothes, I just got my period and they gave me some tampons. I can have a wash* … *I wish I could sleep here!*’ (P11)

Patient factors related to their readiness to change were important in patients' recovery:

‘*I overdosed* ... *that was pretty bad* … *and it shook me up, and I thought it was about time to do something about it. Plus I was sick and tired of getting into trouble trying to raise the money for it.* *’* (P6)

Some patients broke the cycle of multiple exclusion (such as homelessness, offending, and social exclusion) by engaging with drug and alcohol services and tackling the underlying wider determinants of health, including housing. This patient described how his GP had supported his progress:

*‘S* *he has pulled me through from nothing to where I am now. Together and able to keep a flat. And a dog* *.’* (P 5)

The long history of exclusion and sense of fatalism were present:

*‘* *Before being in prison I was on the run for 5 years, so I spent time rough sleeping and 3 years sleeping in a tent. And I wasn’t registered with a GP, so I didn’t take any medication. To be honest at that time, it didn’t matter to me if I didn’t wake up in the morning.* *’* (P1)

As well as being a long journey, patients also described a relapsing and remitting course to exclusion, especially when addiction and alcohol problems were involved:

*‘And things can take a really long time. I have been proactively trying to get off drugs since 2003, and I have only just done it. So that’s 15 years since I was 12 years old*.’ (P7)

There were often life events triggering the patient into homelessness:

*‘* *I lost my flat because of rent arrears. I was in care for 11 years, then they gave me a flat but they didn’t teach me how to pay my bills or look after my flat, so they basically set me up to fail* *.* *’* (P8)

#### High quality care

High quality care was demonstrated by staff going ‘above and beyond’:

*‘* *Yeah, the text messages show they care about you. It’s only a little text but things like that, the food vouchers. Things like that. They care about you.* *’* (P6)

### Staff perspectives

Interviews were conducted with 4 members of staff which included one GP, one practice nurse, one support worker, and one administrator.

#### Ease of access

The impact of literacy and challenging behaviour on patients’ ability to access care was recognised by staff:

*‘* *A number of our patients are illiterate. A lot of our patients would just collapse with any forms, even if they can read and write* *.’* (Staff member [S]4)

The practice therefore facilitates this with a very simplified registration form, and support workers to assist with benefits and housing applications.

#### MDT care

Gaps in the MDT were identified by staff:

*‘* *How wonderful would it to have a podiatrist, an optician, and someone who does dental health. It would be wonderful to have those things here under one roof.* *’* (S1)

#### High quality care

Staff felt that the lack of clinical capacity was impacting on the quality of care, as well as longer waiting times for appointments:

*‘* *There isn’t enough capacity. There are not enough GP hours or nurse prescriber hours. Trying to fit a patient into a small* [time] *slot doesn’t work at all.* *’* (S4)

#### Person-centered care

When asked what progress looked like in this group of patients, increased and meaningful engagement, medication compliance, improved self-care, and motivation were identified:

*‘* *And over the course of a year, from having a deep-seated alcohol issue, he engaged with us less and less but more productively and positively. So success is for instance if they have never attended an appointment, and now attend regular appointments.* *’* (S1)

#### Safety of services

In the domain of keeping services safe, there were mixed opinions from staff:

*‘* *Pe* *ople are angry, people are intoxicated, just lots of vulnerable people, and they all feel safe here, really safe.* *’* (S1)

Other staff felt that the lack of clinical coverage was impacting on safety:

*‘* *I think there are risks to patient’s safety when there no clinicians on site and patients are coming in using showers and the needle exchange, and at risk of overdose in the loos. We are open from 9–5 but we do not provide care from 9-5.* *’* (S4)

#### Reflection and learning

In the domain of commitment to reflection and learning, staff recognised the impact of working within the service on staff:

*‘* *I never say no, which can be really demanding.* *’* (S2)

Given the nature of the work, which can be emotionally demanding, it perhaps attracts conscientious individuals who go above and beyond. It was felt that emotional wellbeing of staff could be improved:

*‘* *There is not enough emotional support for the workers dealing with those stories.* *’* (S2)

#### Service user involvement

There were some gaps in the care provided by the practice, including the lack of service user involvement:

*‘* *We lost some volunteers who were doing the garden, who had a big fallout with the service. It’s really sad, they were doing so well, I think they used to go to conferences and they were trying their hardest to set up something to help other people.* *’* (S2)

Overall, staff were more concerned with clinical safety and lack of support for staff, whereas patients were positive about the service, particularly in the areas of ease of access and person-centred care.

## Discussion

### Summary

Compared to the slope of health inequalities (whereby health problems gradually rise in prevalence with increased deprivation), PEH suffer a disproportionate burden of morbidity, which has been likened to a 'cliff'.^20^

Interviews with patients gave some insight into the reasons why individuals — for whom mental distress, and mental and physical health problems add to already difficult life circumstances — are hesitant about approaching ‘mainstream’ primary care providers. Patient interviews found evidence of the values of continuity of care, ease of access, multidisciplinary care, and person-centred care being achieved. Participants discussed the chronic relapsing and remitting nature of exclusion, and the low self-worth of excluded patients. Staff described concerns with the low levels of patient involvement in the service, lack of opportunities for staff reflection and learning, and low clinical capacity, which were thought to affect service safety and quality.

### Strengths and limitations

The research was conducted in one setting, so care must be taken when transferring the findings to other settings. Owing to the pragmatic approach to interviewing, it was only possible to interview participants as they engaged with the service, thereby missing the views of those who did not attend appointments.

The role of the lead investigator (being both a GP within the practice and a researcher) was fully considered prior to the study so that advantages were augmented and potential disadvantages minimised.^21^ Working within the service gave her prior knowledge and enabled trust and rapport with the patients and staff, thus facilitating recruitment. She did not conduct interviews with patients she had consulted with clinically, but it must be acknowledged that her position as a GP may have influenced power dynamics in the interview. This could cause some social desirability bias,^18^ with patients who had more positive experiences being more likely to participate and alter their account to please the interviewer. She made her role as researcher clear and distinct from her role as GP for the interviews.

The major strength of this study is the sample of patients, who are very often excluded from research^22^ and thus provide unique insight into the nature of exclusion. Trying to capture the experience and insights of both staff and patients adds an interesting and valuable dimension to the research, which has not been shown before.^5^

### Comparison with existing literature

There is evidence in the literature to support the use of the standards. Previous studies have indicated that patients prioritise issues other than health,^23^ such as basic needs of survival like food and shelter. This reflects the findings of a previous study,^24^ which found that homelessness and mental ill health impaired ability to do practical and simple things, such as attend appointments and complete forms; professionals were most successful when they adapted to these circumstances. This supports ease of access as a core value.

Trauma and adverse childhood experiences (ACE) such as abuse are common in this population, as shown by characteristics of those interviewed in this study (Table 1) and previous studies.^25^ A recent UK study suggests that those with four ACEs are twice as likely to binge drink, seven times more likely to be involved in recent violence, 11 times more likely to have been incarcerated, and 11 times more likely to use heroin or crack cocaine.^25^ ACEs and trauma influence patients' ability to regulate emotions, cope with challenges, and engage with care.^25^ This study showed that both staff and patients valued the tolerance and understanding of the complex interplay between emotion, behaviour, and history of trauma, and worked within it to reduce the cycle of repeated rejection and make the service safer.

A previous UK-based study examined how health professionals working in primary care services for PEH viewed their patients' engagement with health care, and explored how these views influenced their practice.^23^ Themes which positively enhanced engagement included a sense of belonging and contributing to a homeless healthcare community. This idea is supported by other studies.^26^ Another study found evidence for person-centred, non-restrictive, flexible programme policies which were respectful to clients' needs, providing stability and consistency to the user.^27^ It also emphasised client choice in decision-making resulting in improved mental health outcomes; that is, a person-centred approach.^27^

A qualitative study of the experiences of PEH as patients demonstrated that homelessness, although an event, is also a gradual process, hence continuity of care is vital. The finding that PEH have low self-worth and a sense of ‘fatalism’ about their health is backed up by previous studies.^23^ The finding that going ‘above and beyond’ helps to alleviate these problems is also supported by literature.^28^ ‘Acts of kindness’ by staff, including *‘treating participants with warmth and humanity and/or mak* *[* *ing* *]* *extra efforts on their behalf’*,^27^ made a real difference to clients’ mental health and helped to engage and retain clients in programmes. Treating patients as people with compassion is vital.^26^

### Implications for research and practice

The FIH framework of values can guide service providers, commissioners, and staff in provision of services, service evaluation, and quality improvement. As an example, service user involvement and greater emotional support for staff are to be developed within this service.

The learning from this study can support clinicians to understand how to deliver primary care to excluded patients, such as reducing barriers to access, treating patients in a non-judgmental way, and placing importance on continuity of care. This study demonstrates that there is a role for specialist primary care services, as patients may find it difficult to access the care they need in mainstream services. For example, successful negotiation with the clinician of a mutually acceptable understanding of the problem for marginalised patients may take more time than is usually available in the general practice consultation.^29^ Staff going ‘above and beyond’, tolerating behaviour, and attending to patients' basic needs helps patient satisfaction and engagement.
